# Methionine metabolism in chronic liver diseases: an update on molecular mechanism and therapeutic implication

**DOI:** 10.1038/s41392-020-00349-7

**Published:** 2020-12-04

**Authors:** Zhanghao Li, Feixia Wang, Baoyu Liang, Ying Su, Sumin Sun, Siwei Xia, Jiangjuan Shao, Zili Zhang, Min Hong, Feng Zhang, Shizhong Zheng

**Affiliations:** 1 Department of Pharmacology, Jiangsu Key Laboratory for Pharmacology and Safety Evaluation of Chinese Materia Medica, Nanjing University of Chinese Medicine, Nanjing, 210023 China; 2 Department of Pharmacology, Jiangsu Key Laboratory of Therapeutic Material of Chinese Medicine, Nanjing University of Chinese Medicine, Nanjing, 210023 China; 3 Department of Pharmacology, State Key Laboratory Cultivation Base for TCM Quality and Efficacy, Nanjing University of Chinese Medicine, Nanjing, 210023 China

**Keywords:** Metabolic engineering, Pathogenesis

## Abstract

As one of the bicyclic metabolic pathways of one-carbon metabolism, methionine metabolism is the pivot linking the folate cycle to the transsulfuration pathway. In addition to being a precursor for glutathione synthesis, and the principal methyl donor for nucleic acid, phospholipid, histone, biogenic amine, and protein methylation, methionine metabolites can participate in polyamine synthesis. Methionine metabolism disorder can aggravate the damage in the pathological state of a disease. In the occurrence and development of chronic liver diseases (CLDs), changes in various components involved in methionine metabolism can affect the pathological state through various mechanisms. A methionine-deficient diet is commonly used for building CLD models. The conversion of key enzymes of methionine metabolism methionine adenosyltransferase (MAT) 1 A and MAT2A/MAT2B is closely related to fibrosis and hepatocellular carcinoma. In vivo and in vitro experiments have shown that by intervening related enzymes or downstream metabolites to interfere with methionine metabolism, the liver injuries could be reduced. Recently, methionine supplementation has gradually attracted the attention of many clinical researchers. Most researchers agree that adequate methionine supplementation can help reduce liver damage. Retrospective analysis of recently conducted relevant studies is of profound significance. This paper reviews the latest achievements related to methionine metabolism and CLD, from molecular mechanisms to clinical research, and provides some insights into the future direction of basic and clinical research.

## Introduction

Chronic liver disease (CLD) represents a significant public health concern worldwide.^1^ Viruses, metabolic dysfunction, autoimmune diseases, and alcoholism are the causes of chronic liver injury, all of which almost causes liver fibrosis.^2,3^ In addition, CLD is an established risk factor for hepatocellular carcinoma (HCC).^4^ Metabolic disorders characterize most liver diseases. Patients with CLD often have alterations in glucose,^5^ trace elements,^6–11^ lipid,^12^ and protein metabolism.^13–15^ The effects of liver diseases on amino acid (AA) metabolism have received widespread attention. Abnormally high concentrations of cysteine, methionine, and aromatic AAs were observed in patients with cirrhosis.^16^ The serum levels of branched-chain AAs decreased in patients with CLD.^13^ Nutritional therapy is used to control and manage liver metabolism, and this may improve liver function and have a positive influence on liver diseases.^17^ A better understanding of nutrient metabolism, in which methionine metabolism plays a vital role, could help identify novel therapeutic targets for preventing CLD.

Methionine, an essential proteogenic AA, is necessary for average growth and development,^18^ and breakdown of methionine in the small intestine generates free methionine. Subsequently, the free methionine is absorbed and used for protein synthesis or is converted to S-adenosylmethionine (SAM/AdoMet).^19^ SAM acts as a major methyl and sulfate group donor in numerous biochemical reactions^20^ and is recommended for the treatment of certain diseases. SAM synthesis is suppressed in CLD, therefore, considerable interest has been focused on utilizing SAM for reducing disease severity (Table 1).^19^ However, clinical research on methionine supplementation remains insufficient and the results are controversial.^21^ Here, we offer an in-depth review of the latest achievements related to the physiological and pathophysiological roles of methionine metabolism in liver diseases, from molecular mechanisms to clinical research. We also provide some recommendations for further research.

**Table 1:** The main CLDs affected by methionine deficiency, and of any of the enzymes that participate in the transsulfuration pathway

Chronic liver disease	Adverse consequences of methionine deficiency	References
Viral hepatitis	Low STAT methylation level, the change of MAT1A/MAT2A and the lower deposition of H3K4me3 on HBV-DNA	^31,57–59^
Alcoholic liver disease	Cystathionine and serum homocysteine elevate. MATα1 level, GSH, folate and vitamin B6, and B12 decrease. Decreased ratio of SAM/SAH directly affects the methylation level, ethanol tampers with multiple enzymes, including MAT, BHMT, and various MTs. The lack of PRMT causes lower PE methylation, which leads to SAM accumulation and sensitivity to oxidative stress.	^73,74,76–78,81–83^
Nonalcoholic fatty liver disease	Hepatic Fgf21 mRNA was increased, which is a modulator of energy homeostasis. FFA accumulates and can cause lipotoxicity through JNK1 activation. CD36 level, the PC/PE ratio, and serum homocysteine increase.	^93,96,98–100^
Liver fibrosis and cirrhosis	The phosphorylation of MATα2 and MATβ proteins enhanced. The binding of E2F-4 to MAT2A promoter attenuates. SAM/SAH ratio and DNA methylation decrease.	^22,113,117^
Hepatocellular carcinoma	GNMT is downregulated, MAT1A expression decreases while MAT2A increases. The activity of ODC increases. High levels of CBS express in HCC, which involve in cell proliferation. The expression of SAHH/AHCY is inhibited.	^48,109,131,138,152^

## The physiological role of methionine metabolism

**Fig. 1:**
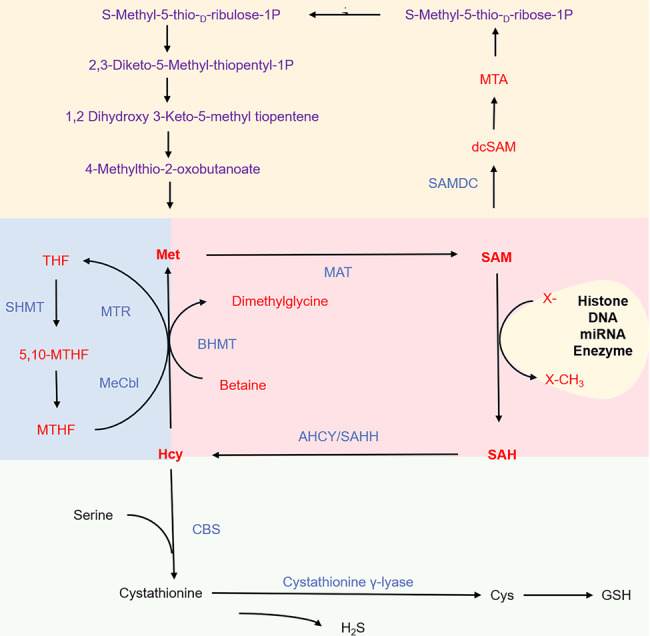
Response of methionine metabolism in the liver. There are four main participants in this pathway, namely methionine, S-adenosylmethionine (SAM), S-adenosyl homocysteine (SAH), and homocysteine (Hcy). Methionine adenosyltransferase (MAT) converts methionine to SAM and then uses a methyl donor catalyzed methyl donor. Another product of these reactions is SAH, which is reduced by S-adenosine homocysteine protease (AHCY/SAHH) to adenosine and Hcy. Methionine metabolism involves the folate cycle, the transsulfuration pathway, and the salvage pathway. AHCY adenosylhomocysteinase, BHMT betaine homocysteine methyltransferase; GSH glutathione; Hcy homocysteine, SAM S-adenosylmethionine, SAH S-adenosyl homocysteine, Met methionine, MTs methyltransferase, CBS cystathionine-β-synthase, Cbl cobalamin, vitamin B12, MeCbl methylcobalamin, MTA 5′‐methylthioadenosine, dcSAM decarboxylated SAM, MTHFR methylenetetrahydrofolate reductase, SHMT serine hydroxymethyltransferase

Methionine metabolism can be divided into the methionine cycle, transsulfuration pathway, and salvage cycle (Fig. 1). First, methionine adenosyltransferases (MATs) catalyze the biosynthesis of SAM from methionine and ATP.^18,22^ Under the catalysis of methyltransferases (MTs), SAM donates its methyl group for methylation and converts itself to S-adenosyl-homocysteine (SAH).^23,24^ SAH is then converted by S-adenosyl-homocysteine hydrolase (SAHH/AHCY) to homocysteine (Hcy).^25^ Hcy promotes glutathione (GSH) synthesis by entering the transsulfuration pathway or is converted back to methionine by methionine synthase (MTR/MS), accordingly completing the methionine cycle. MTR requires the methylated form of vitamin B12 (cobalamin) and uses 5-methyltetrahydrofolate, as a methyl donor for catalysing Hcy remethylation.^24^ Parallel to this process, betaine homocysteine methyltransferase (BHMT) can catalyze the formation of SAM from the methyl donor betaine.^25,26^ In the transsulfuration pathway, cystathionine-β-synthase (CBS) catalyzes cystathionine synthesis through the condensation of Hcy and serine. Then, cystathionine-γ-lyase hydrolyzes cystathionine to produce cysteine for GSH synthesis. In addition, cystathionine-γ-lyase and CBS catalyze the production of hydrogen sulfide in these processes.^18^ Methionine can also be recovered from methylthioadenosine (MTA), a by-product of polyamine (PA) biosynthesis, via the methionine salvage pathway. Furthermore, SAM is decarboxylated by AdoMet decarboxylase to form decarboxylated SAM (dcSAM). After donating of an aminopropyl group for PA synthesis, dcSAM transforms to MTA, which is then converted back to methionine via six enzymatic steps.^27–29^

SAM is the principal methyl donor for the methylation of phospholipids, nucleic acids, and biogenic amines.^19^ It is likewise the second most common enzymatic cofactor after ATP.^30^ Methionine concentration is closely related to the ratio of SAM to SAH, which affects many methylation reactions, including histone methylation.^31,32^ Alterations in the methylation status contribute to many pathophysiological conditions, including cancer, obesity, and ageing.^33,34^ Methionine is among the major targets of reactive oxygen species (ROS).^35–37^ Low methionine concentration can also lead to cell morphological changes and cell proliferation.^31^ Maladjustment of methionine metabolism, which plays a crucial role in cellular physiology, occurs in sundry diseases.^18^ Liver lesions are closely related to profound alterations in methionine metabolism.^38^ Copeland et al. linked the alterations in the methionine cycle and liver injury for the first time.^39^ A systematic review reported that SAM improves some liver biochemical parameters and symptoms in patients with intrahepatic cholestasis,^40^ which is a feature of several CLDs.^41^ Furthermore, in recent years, *N*^*6*^-methyladenosine (m^6^A) modifications have been proven to be related to liver injuries. Deregulation of m^6^A regulators in host hepatocytes may contribute to the development of viral hepatitis.^42^ Methionine metabolism is closely related to m^6^A methylation.^42,43^ A study revealed a mechanism of homeostatic regulation of SAM synthesis in mammalian cells that involves dynamic m^6^A modifications in the MAT2A 3′ UTR.^44^ After methionine depletion, splicing of the MAT2A-retained intron is rapidly induced.^45^

**Fig. 2:**
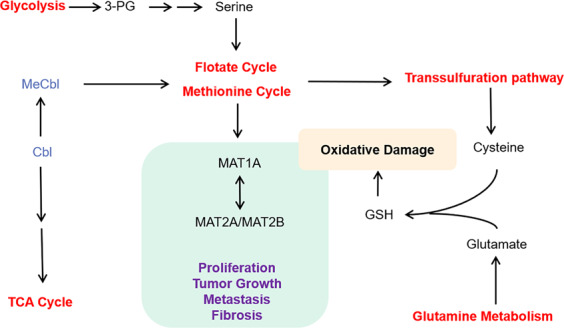
Cross talk between methionine metabolism and the other metabolism. Glycolysis produces ATP and 3-phosphoglycerate (3-PG), which are used in serine synthesis and folate cycle. ATP can be used to transform methionine into SAM. Cobalamin (Cbl) is closely related to TCA, while the methylcobalamin (MeCbl) is related to the folate cycle and methionine cycle. Glutamic acid produced by glutamine metabolism can be used in the synthesis of GSH. 3-PG 3-phosphoglycerate, Cbl cobalamin, vitamin B12, MeCbl methylcobalamin, TCA cycle tricarboxylic acid cycle

Methionine metabolism is closely related to various metabolic pathways (Fig. 2). The folate cycle, coupled with the methionine cycle, constitutes a double ring metabolic pathway. All such bicyclic pathways are collectively referred to as one-carbon metabolism.^46,47^ Methionine metabolism is the pivot linking the folate cycle to the transsulfuration pathway. The intermediate Hcy, which is a sulfur-containing, nonprotein, toxic AA,^48^ connects the transsulfuration pathway with the methionine cycle. Hcy clearance is essential for genetic protection.^25^ As the first enzyme in the transsulfuration pathway, CBS has a SAM regulatory site and mutation at this site results in homocystinuria,^30^ which is closely associated with cancer.^48^ GSH synthesis also links the transsulfuration pathway with glutamine metabolism, which upregulated by many oncogenic insults and mutations.^49^ The gene for 5-methylthioadenosine phosphorylase (MTAP), a key enzyme of the methionine salvage pathway, is frequently deleted in human cancers.^50,51^ Inhibition of protein arginine N-methyltransferase 5 (PRMT5) has recently emerged as a potential therapy against MTAP-deficient cancers.^52^ MTA inhibits PRMT5 by competing with SAM for binding to the catalytic site.^50^ Methionine restriction (MR) is sufficient for eliminating MTA accumulation to levels found in MTAP-expressing cells.^53^ Besides, methionine metabolism is closely related to PA metabolism. An increased in PA levels caused by ornithine decarboxylase (ODC) activation may lead to the pro- or anti-inflammatory roles of PAs.^29^ Thus, methionine metabolism plays an important role in various biological metabolisms.

## Methionine metabolism and CLDs

### Role of methionine metabolism in viral hepatitis

Hepatitis B virus (HBV)^54^ and hepatitis C virus (HCV) cause chronic liver injury.^55^ Significant ethnic differences are observed in viral infection rates.^55^ HCV infection can downregulate protein arginine N-methyltransferase 1 (PRMT1) through protein phosphatase 2A (PP2A), while PRMT1 can catalyze STAT-1 methylation on Arg 31.^56^ STAT-1 is a transcription factor that participates in viral signaling responses and responses to interferon (IFN) signalling activation.^57^ Duong et al.^58,59^ demonstrated that downregulation of IFN-sensitive gene expression by interference with STAT-1 methylation can promote interaction with the protein inhibitor of activated STATs (PIAS). After SAM administration, the antiviral effect of IFN was enhanced.^59^ Feld et al. also showed that adding SAM to peginterferon (PEG-IFN) and ribavirin improves the kinetics of the early antiviral response.^60^ Sonia Amelia Lozano-Sepulveda et al. suggested that SAM can diminish HCV expression in cells partly by modulating antioxidant enzymes, synthesizing GSH, and switching the MAT1A/MAT2A turnover.^57^ The MAT1A/MAT2A ratio is relevant to the survival of patients with HCC.^61^ This transformation may be conducive to the transition of viral hepatitis to liver cancer. SAM can balance the MAT1A/MAT2A ratio, which may reduce or even prevent the further development of the disease. Furthermore, in a HCV study involving ethanol feeding, betaine treatment attenuated the damage caused by ethanol metabolism to STAT-1 methylation.^62^ However, in a phase II randomized controlled trial, SAM neither reduced liver damage in patients with HCV cirrhosis nor improved the liver function.^21^ Considering the pharmacokinetics of SAM, the high chemical reactivity of the methyl group of SAM and its spontaneous decomposition may lead to adverse effects. Thus, the efficacy and safety of SAM against HCV need to be investigated in further studies.^63^

**Fig. 3:**
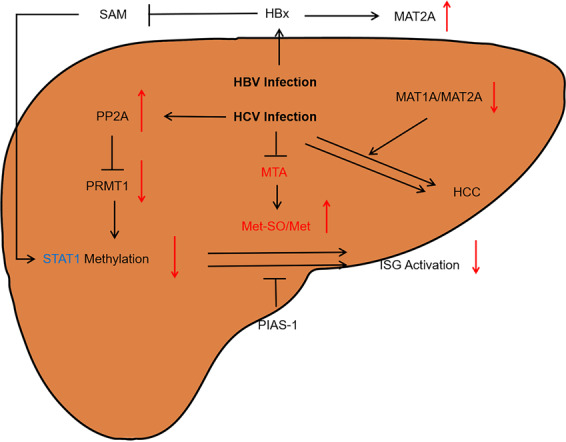
The metabolism of methionine in viral infections of hepatitis B and C. The hepatic polyamine synthesis and transsulfuration pathway activities are impaired in virus infection. Methylthioadenosine (MTA) is a sulfur-containing adenine nucleoside produced from SAM during the synthesis of polyamines, including spermine and spermidine. The level of MTA significantly decreases during the late stage of HCV infection in cells. Moreover, Met is particularly susceptible to elevated ROS levels. Upon reacting to ROS, protein-bound Met is readily oxidized to form methionine sulfoxide (Met-SO). The increased Met-SO level and Met-SO/Met ratio indicate increased oxidative stress subsequent to decrease liver function. Furthermore, the STAT-1 genes showed significant difference between HBV and HCV. Met methionine, Met-SO methionine sulfoxide, HBx the X protein of HBV, ISG interferon-stimulated gene, PP2A protein phosphatase 2A, PRMT1 protein arginine methyltransferase 1, PIAS protein inhibitor of activated STATs

Current therapies for chronic hepatitis B (CHB) mainly use PEG-IFN and orally administered nucleotide analogs.^55^ Deposition of H3K4me3 on HBV-DNA was reduced in HBV early antigen negative stage samples from CHB patients, which correlated with the levels of viral transcripts,^64^ while the H3K4me3 signature is modulated in response to a decrease in SAM and SAH levels.^31^ Liu et al. demonstrated that the X protein of HBV enhances the binding of transcription factors NF-κB and cAMP-response element-binding protein to the promoter of the *MAT2A* gene, and thus regulates its expression, which is essential in HBV-mediated HCC progression.^65^ Guo et al. showed that SAM concentrations are related to the severity of HBV-related liver disease.^66^ HBV inhibited STAT-1 methylation dramatically. Combined with IFN-α, SAM treatment effectively improved STAT-1 methylation and attenuated STAT-1 - PIAS1 binding.^67^ In establishing a practical cure for chronic HBV infection, the significance of T cells has been confirmed.^68^ Sinclair et al. showed that a steady supply of methionine is required for T cells to remain activated, and T cell activation increases the demand for methionine.^69^ Bing et al. found that the combination therapy of glucocorticoids, SAM, and IFN-α is possibly useful for CHB patients.^70^ HBV CpG methylation is closely linked to hepatocarcinogenesis. DNA methylation also plays a major role by silencing tumor suppressors in HCV-infected patients with HCC. Between HBV and HCV samples, in terms of both expression and methylation levels, researchers found that the greatest differences were three genes: human leukocyte antigen, STAT-1, and 2′5′oligoadenylate synthetase 2. The in-depth study of methylation differential genes will help elucidate diverse mechanisms of HBV and HCV pathogenesis, and further benefit antiviral therapies.^71^ Considering the important role of the methionine cycle in methylation, the relationship between the methionine cycle and HBV and HCV at the molecular level needs further exploration (Fig. 3). The role of methionine metabolism in virus invasion needs additional research.

### Role of methionine metabolism in alcoholic liver disease

Prolonged exposure to ethanol causes sustained and noticeable liver damage,^72^ from steatosis to alcoholic steatohepatitis to fibrosis, even cirrhosis.^23^ Alteration in methionine metabolism plays a vital role in the development of alcoholic liver injury.^23,73^ Long-term ethanol consumption results in increased Hcy and SAH levels. The increase in SAH levels is sufficient for sensitizing the liver and hepatocytes to tumor necrosis factor (TNF) cytotoxicity.^74^ The development of steatosis and the inhibition of proteasome activity, both hallmark signatures of alcohol-induced liver damage, occur as a result of the reduced SAM/SAH ratio.^75^ The decreased SAM/SAH ratio directly affects the methylation level.^74^ Ethanol tampers with the function of multiple enzymes, including MAT, BHMT, and various MTs.^76,77^ Decreased MAT activity is attributable to alcohol-induced oxidative stress and reactive aldehydes, which can inactivate the liver-specific MAT. The lack of metabolic products due to impaired methionine metabolism inhibits remethylation of Hcy, the form of GSH, thus weakening defenses against oxidative stress.^23,78^ The increase in SAH levels and hypomethylation may severely impact the expression/activity of caspase-8, which correlates with enhanced apoptosis of alcoholic liver disease (ALD).^79,80^ Furthermore, individuals with alcoholic hepatitis have lowered levels of MATα1, which directly interacts with CYP2E1 and facilitates CYP2E1 methylation at a critical arginine residue.^81^ Besides, SAM can participate in lipid synthesis through the phosphotidylethanolamine N-methyltransferase pathway, which is essential for forming very-low-density lipoproteins (VLDLs).^82^ Lack of phosphatidylethanolamine (PE) methylation leads to the SAM accumulation, which results in hypermethylation of histones and the major phosphatase PP2A, dependency on cysteine, and sensitivity to oxidative stress.^83^

By activating the Nrf2-ARE pathway, methionine availability promotes endogenous antioxidant responses and plays a key role in inhibiting ROS-induced oxidative stress.^84^ In various experimental models of liver diseases, both SAM and betaine attenuated ethanol-induced liver injury.^23,75^ SAM supplementation reverses the depletion of SAM and GSH in ethanol-fed animals, and restores the fluidity of the mitochondrial inner-membrane. In addition, it alleviates steatosis and hepatocyte necrosis.^19^ The marked impact of SAM supplementation is prevention of mitochondrial DNA damage and mitoribosome dissociation.^85^ SAM attenuates injuries by regulating the metabolism of beneficial cytokines.^74^ SAM also downregulates potentially toxic pro-inflammatory cytokines.^79^ Betaine per se does not directly interact with oxidants. In the methionine cycle, it mainly mediates SAM synthesis,^86^ and thus restores the SAM/SAH ratio, and recovers DNA methylation and gene expression.^87^ Betaine also alleviates alcohol-induced free fatty acid (FFA) accumulation by correcting an alcohol-induced imbalance in fatty acid (FA) synthesis and oxidation by targeting hepatic sterol regulatory element-binding protein (SREBP)-1c, FA synthase, and peroxisome proliferator-activated receptor γ (PPARγ) coactivator 1α.^88^ SAM exhibits direct antioxidant activity by scavenging ROS.^86^ In clinical trials, SAM increases the levels of the cellular antioxidant GSH in patients with ALD, and the survival of patients with less advanced liver cirrhosis improves with SAM.^89,90^ Prednisolone plus SAM can produce an improved therapeutic response.^91^ The maladjustment of methionine and SAM metabolism has been well-accepted in ALD, while the effect of this maladjustment on downstream products has not been fully investigated.^79^ Multiple pieces of evidence have linked ethanol-induced abnormal methionine metabolism to deficiencies of folate, and vitamin B6 and B12, which are key factors in ALD pathogenesis.^73^ The trial of SAM treatment for ALD is inconclusive. Larger and longer-term clinical trials are needed, and supplementation with other compounds important for methionine metabolism, such as vitamin B6, should be considered in ALD patients. Betaine should also include be investigated as a supplement in large-scale clinical studies.^90^

### Role of methionine metabolism in nonalcoholic fatty liver disease

Nonalcoholic fatty liver disease (NAFLD) is a result of defects in multiple metabolic pathways leading to the accumulation of triglycerides (TGs) in the liver.^92^ NAFLD frequently progresses to nonalcoholic steatohepatitis (NASH), which is a result of prolonged inflammation and hepatocyte damage, ultimately causing fibrosis, HCC, and even death.^93,94^ Human NASH is associated with hypomethylation of liver DNA.^92^ A previous study showed a lower rate of methionine transmethylation in insulin-resistant patients with NASH.^95^ Gene deletion or the lack of unstable methyl groups in the form of methionine and choline undermines the ability to synthesize SAM, which leads to the development of steatosis and its rapid progress toward NASH.^96,97^ Treatment with a methionine and choline-deficient diet (MCD) is a routine, and useful method for inducing NASH in rodents.^93^ Saturated FFA accumulates in MCD-fed mice and can cause lipotoxicity through JNK1 activation, leading to mitochondrial damage and ROS production.^98^ Notably, MAT1A-KO mice exhibit chronic liver SAM deficiency, and spontaneously develop steatohepatitis and HCC.^81^ The liver of these mice have high TGs, diglycerides, FAs, and ceramide. CD36 content significantly augments in MAT1A*-*KO mice.^92^ Increasing the CD36 level contributes to hepatic TG storage.^99^ Besides, the levels of Hcy, an intermediary in liver methionine metabolism, are elevated in patients with NAFLD.^100^ Elevated serum Hcy concentrations are related to the histological severity of NAFLD.^101^ However, high serum Hcy levels are negatively associated with NASH, and significant fibrosis in patients with NAFLD. The pathophysiological mechanisms between Hcy and NAFLD are multifactorial, and not fully understood.^102^

SAM is the key methyl donor for phosphatidylcholine (PC) synthesis, which is required for the export of VLDLs from the liver.^95^ Beyond its role as a methyl donor, SAM can act as a metabolic regulator, controlling processes, such as regeneration, differentiation, and organ sensitivity to different injuries.^103^ Mitochondrial polarization was restored in MAT1A-KO hepatocytes upon incubation with SAM.^92^ Adding methionine to a high-cholesterol diet can significantly reduce hepatic steatosis (HS), oxidative stress, and fibrosis caused by high choline alone.^104^ However, due to the lack of early diagnosis, conducting human research in the initial stage of NASH is impossible. Moreover, Maria del Bas et al. found that selenium and vitamin E deficiency together cause an increase in SAM, and suppress MYC expression in livers of hamsters on a high-fat diet, which accelerated the development of hamster NASH. Therefore, under certain circumstances, increasing hepatic SAM might not be an efficient strategy.^103^ Similarly, Yao et al. showed that excess SAM is harmful. A high-methionine diet (HMD) caused hyperhomocysteinemia (HHcy) and HS in mice.^99^ The association between HHcy and NAFLD has been well investigated.^104,105^ Conversely, reducing or limiting methionine intake has beneficial effects, such as increased energy expenditure, extended lifespan, improved insulin sensitivity, and reduced adiposity.^106–108^ Dietary MR can substantially lower circulating levels of methionine in SAHH-deficient patients.^109^ Gao et al. showed that dietary MR can induce particular metabolic profiles rapidly in clinical settings.^110^ According to some studies, methionine induces hypercholesterolemia by facilitating choline synthesis in the liver. Nevertheless, sitagliptin in conjunction with a high-choline diet exacerbates oxidative damage, leading to symptoms that are similar to those of NASH, whereas the HMD will partially attenuate the negative effects.^104^ Therefore, whether NAFLD patients need a large amount of SAM supplementation needs to be investigated. Furthermore, methionine and intermediates formed during its metabolism may play a separate or synergistic role, thereby conferring hepatoprotective effects; however, this needs to be explored further.

### Role of methionine metabolism in liver fibrosis and cirrhosis

Liver fibrosis is a characteristic of almost all CLDs and remains a crucial determinant of clinical prognosis.^1^ In CLD, the imbalance between the new deposition of the extracellular matrix and its resorption leads to the development of fibrosis, indicating the liver’ s response to repeated wound healing. Eventually, it may lead to cirrhosis.^111^ Hepatic fibrosis can be considered bidirectional dynamic development and regression. Activated hepatic stellate cells (HSCs) are considered a key factor in fibrosis pathogenesis.^112,113^ Two MAT genes, MAT2A and MAT2B, are required for HSC activation. For the first time, Ramani et al. identified that the phosphorylation of MATα2 and MATβ proteins is enhanced during HSC activation. The stability of these proteins favors human HSC trans-differentiation.^22^ *MAT2B* affects HSC activation through ERK and PI-3K signalling mechanisms, whereas MAT2A affects the growth of HSCs by influencing the changes in SAM levels.^113^ In quiescent HSCs, PPARγ is a negative regulator of MAT2A. A transition from the quiescence state to the activation state abolishes this control and permits PPARβ to increase MAT2A transcription.^114^ Besides, leptin-induced multiple signalling pathways mediate HSC activation.^115^ MAT2B-KO completely blocks leptin-mediated induction of STAT3 phosphorylation.^116^ A recent study showed that leptin-induced β-catenin signalling attenuats E2F-4 binding to the *MAT2A* promoter, thus increasing its activity.^117^ HSC activation causes a decline in SAM and MTA levels, a decrease in the SAM/SAH ratio, and overall hypomethylation of DNA.^113^ The decrease in SAM levels is related to lower MAT II activity during activation.^118^

Ramani et al. revealed that mutations in the phosphorylation sites of Y371/Y374 in MATα2, and T257/Y259 in MATβ inhibit HSC activation.^22^ Mutation of specific phosphorylation sites may be used as a strategy for treating fibrosis and even liver cirrhosis caused by HSC activation. A previous meta-analysis confirmed the protective effects and safety of SAM for CLD. Animal experiments have shown that depletion of SAM level is related to liver fibrosis.^66^ In an ethanol-LPS fibrotic liver rat model, SAM addition inhibits oxidative stress and HSC activation, thereby significantly reducing liver damage and fibrosis.^119^ Pharmacological doses of SAM and MTA can downregulate the expression of MAT genes and interrupt leptin-mediated signalling.^116^ SAM significantly inhibited type I collagen secretion and increased NF-κB activity. SAM also increased type I collagen polyubiquitination.^120^ Furthermore, SAM inhibits HSC contraction by interfering with the formation of F-actin stress fibers and phosphorylated myosin light chains.^121^ SAM act as potent inhibitors of Wnt signalling and Wnt-induced lysosomal extracellular protein digestion.^122,123^ Wnt/β-catenin signalling is a major therapeutic target for liver fibrosis.^124^ A recent study found that intracellular SAM concentration is regulated by the TGF-β1/p65/MAT2A signalling pathway, and may be targeted in liver fibrosis treatment.^125^ Studies regarding SAM and reversion of liver fibrosis are still lacking. Liver sinusoidal endothelial cells (LSECs), which maintain liver homeostasis, as well as HSC and Kupffer cell quiescence, are the main players in resolving fibrosis.^126^ Exploration of the relationship between methionine metabolism, LSECs, and fibrosis regression may provide unexpected findings.

### Role of methionine metabolism in HCC

Globally, HCC is the fourth most common cause of cancer-related death.^127,128^ Surgery remains the most effective treatment with curative potential, and novel treatments are urgently needed.^66^ MAT1A is a marker for normal liver differentiation, and the expression of MAT2A and MAT2B increases during rapid liver growth and dedifferentiation.^129,130^ During the general development of the fetal liver, the originally expressed MAT2A is gradually replaced by MAT1A.^116,131^ MAT2A and its gene product, MAT IIα (dimer formed from α2 subunits), are overexpressed in various human epithelial tumors. TNF-α upregulates MAT2A via NF-κB and adaptor-related protein complex 1.^132^ MAT2A overexpression improves the activity of the V-Maf Avian Musculoaponeurotic Fibrosarcoma Oncogene Homolog G (MAFG) promoter. High MAFG expression correlates with tumor progression and reduced survival time.^61^ Liu et al. revealed that in liver cancer, hypoxia activates MAT2A expression through hypoxia-inducible factor-1α, resulting in increased MAT II enzyme activity and reduced SAM production, which then induces genomic DNA demethylation.^133^ Tumor cell proliferation is inhibited by histone acetylation, which promotes MAT IIα ubiquitylation and subsequent proteasomal degradation.^132^ Inhibition of MAT1A expression leads to tumor growth, invasion, and metastasis.^130^ A recent study showed that upregulation of forkhead box M1 decreases MAT1A, while raises NF-κB expression; thus, forming a feed-forward loop that enhances tumorigenesis.^134^ Prohibitin 1 (PHB1) and MAT1A positively regulate each other, while PHB1 and MAT1A mutually regulate c-MYC/MAFG/c-MAF.^135^ Silencing of MAT2B could remarkably inhibit migration and invasion.^136^ A cross talk between MAT2B and HuR and SIRT1 protein influences the therapeutic effect of resveratrol on liver cancer cells.^137^ Moreover, glycine N-methyltransferase (GNMT), which is the most abundant liver MT regulating the availability of SAM, is downregulated in HCC. Deletion of the gene for GNMT promotes a shift in metabolism and the transfer of nutrients from glucose formation to utilization of elevated levels of SAM. ^138^ Furthermore, MCD, as a commonly used model for inducing CLD and even HCC, is closely link to cancer.^139–142^ Choline depletion affects lipid metabolism and transport.^143^ Jiang et al. found that choline supplementation increases global DNA methylation and the expression of peroxisomal acyl-coenzyme A oxidase 1, which mediates FA β-oxidation.^144^ Liu et al. showed that higher choline intake can improve the overall general health.^145^ In juvenile black seabream, choline supplementation suppressed NF-κB activation and increased the expression of lipolysis pathway genes.^146^ The increased uptake of choline by HCCs cells promotes phospholipid formation, DNA hypermethylation, and hepatocyte proliferation. Gougelet et al. showed that choline-deficient diet reverses these effects and promotes regression of HCC that overexpress β-catenin in mice.^147^ Some controversies regarding MR do exist. Limited intake of methionine attenuates steatosis and delays the development of NASH through various signal transduction pathways and effector molecules, including SREBPs, sirtuins, and the growth hormone/insulin-like growth factor-1 axis.^148^ Dietary MR and cysteine restriction have beneficial effects on circulating biomarkers, including FGF21,^149^ and MR protects against metabolic diseases and ageing, represses cancer development, and improves cancer therapy.^150,151^ However, MAT1A-KO mice, characterized by chronic SAM deficiency, exhibit macrovesicular steatosis, mononuclear cell infiltration in periportal areas, and HCC development.^38^ MR leads to insufficient SAM, leading to MAT1A/MAT2A transition and the overall DNA hypomethylation, decreased DNA reduction, and genomic instability and abnormal signal transduction are related, including c-MYC overexpression, increased PA synthesis, RAS/ERK, PI-3K upregulated/AKT, and LKB1/AMPK axis. The decrease in SAM levels leads to HCC cell proliferations, cell survival, and microvascular formation.^96^

Reduced SAM levels and dysregulation of MATs are considered potential therapeutic targets for HCC. Early studies have shown that in exogenous SAM-treated rats, ODC activity, and PA synthesis are significantly reduced in preneoplastic liver lesions.^152^ When mice are treated with SAM or ursodeoxycholic acid, MAFG induction is weakened during bile duct ligation. When a combination of these drugs are administered, MAFG induction is completely blocked.^61^ When MAT1A expression increases, the LIN28B promoter region becomes highly methylated, increasing the expression of let-7.^130^ Increasing MAT1A expression seems to be an effective treatment strategy. Overexpressing *MAT1A* in the Huh7 cell line steadily increased SAM levels and cell apoptosis, decreased cell growth, and decreased the expression of angiogenesis genes. MATα1 also interacts with p53 and DNA damage-regulated gene 1 in hepatoma cells.^129^ Furthermore, SAM treatment altered the homeostasis of MAT1A and MAT2A by altering the balance of AUF1 and methyl-HuR/HuR, which was first identified to inhibit MAT2A mRNA stability.^153^ SAM maintains MAT1A expression, but inhibits MAT2A expression, in hepatocytes. Pharmacological doses of SAM and its metabolite MTA promote apoptosis of liver cancer cells, while resisting the apoptosis of normal liver cells;^116^ thus, SAM is an attractive chemopreventive agent.^129^ Exogenous SAM can inhibit HCC development by recovering the normal level of SAM.^131^ However, 24-day intravenous infusion of SAM did not affect the size of the formed tumors, which may be due to the compensatory induction of GNMT, and prevented SAM accumulation.^38^ A study revealed that tumor-initiating cells become addicted to exogenous methionine because of highly elevated methionine cycle activity. Even transient inhibition of the methionine cycle is sufficient to weaken the tumor-initiating capability.^154^ Treatment effectiveness of MR is dependent on many factors. More studies are warranted to determine the regulatory effects.^155^ SAM showed a meaningful clinical value for patients with advanced tumors and improved prognosis; however, the efficacy of SAM treatment needs further exploration in randomized prospective clinical trials.^66^ In addition, glutamine metabolism is a hot research topic.^156^ Local glutamine deficiency promotes tumor dedifferentiation by inhibiting histone demethylation.^157^ Glutamine controls ROS through GSH synthesis.^49^ Glutamine metabolism relies on the methionine cycle in one-carbon metabolism to exert its anti-ROS function. Compared with glutamine metabolism, methionine metabolism is less studied, and thus is worthy of further research. Meanwhile, targeting multiple metabolic pathways to suppress the tumor growth is the best treatment strategy.^156^

## Conclusion

In conclusion, as an important part of one-carbon metabolism, methionine metabolism is closely related to diverse pathophysiological processes.^19^ Accumulating preclinical evidence indicates that alterations in the methionine cycle play a pathogenetic role in CLD.^38^ The switch of MAT1A to MAT2A/MAT2B reduces the levels of SAM, which is an essential factor in fibrosis and liver cancer.^129^ Preventing or even reversing this transformation will be the direction of future research. Controlling SAM levels precisely for liver injury is important, but SAM regulation is not well understood. A previous study showed that METTL16 is associated with SAM homeostasis.^45^ Future studies can focus on the relationship between methionine metabolism and m^6^A. In clinical research, whether methionine supplementation is necessary for CLD remains controversial. According to the existing research, methionine supplementation can be combined with basic clinical drugs; and this may have unexpected results. High-quality, prospective clinical trials are required to prove or refute the benefit of SAM supplementation.^19^ Moreover, SAM is transported across the intestinal epithelium by a strictly paracellular mechanism in the absence of membrane transporters. As a highly polar molecule, SAM is not likely to penetrate lipid membranes. Several studies have reported that both Caco-2 cells and hepatocytes exhibit very low uptake of SAM.^158^ In mammalian cells, transport of SAM appears to occur exclusively in brain endothelia, but not in non-pathological cells of the periphery.^159^ Exogenous SAM does not penetrate the plasma membrane, but equilibrates with a small sucrose-inaccessible compartment on the outer side of this membrane.^160^ These studies thus have indicated that in the treatment of CLD, the impact of SAM supplementation may not be direct. Studies have shown that exogenous SAM is utilized for phospholipid methylation on the outer surface of the plasma membrane.^161^ Exogenous SAM-mediated control of DNA methylation and gene expression could be a mechanism of the SAM anti-progression effect.^162^ The mechanisms of intestinal absorption and hepatic uptake of exogenously administered SAM, and the mechanism of its hepatoprotection remain unknown.^158^ SAM supplementation reduces CLD severity.^40^ This molecular mechanism is closely related to the role of SAM participating in methylation reactions to provide methyl groups, entering the transsulfuration pathway to metabolize and synthesize GSH, and participating in the one-carbon cycle. However, not many studies have investigated how exogenous SAM intervenes in intracellular metabolism, and additional studies are warranted.

## Supplementary information

Polish certificate
