# Supplementary material for: Methionine metabolism in chronic liver diseases: an update on molecular mechanism and therapeutic implication
Source: Signal Transduct Target Ther. 2020 Dec 4;5:280. doi: 10.1038/s41392-020-00349-7 (PMC7714782; doi:10.1038/s41392-020-00349-7)
Supplement: Supplementary file 1 — Polish certificate [file 41392_2020_349_MOESM1_ESM.pdf]

尚辑 Sagesci

SCI科研服务品牌

CERTIFICATE OF ENGLISH EDITING

This is to certify that the manuscript entitled

Methionine metabolism in chronic liver diseases: an update on molecular mechanism and therapeutic implication

By:Zhanghao Li

commissioned to us has been carefully edited by a native English-speaking editor of sagesci, and the grammar, spelling, and punctuation have been verified and corrected where needed. Based on this review, we believe that the language in this paper meets academic journal requirements. Please contact us with any questions

Date of issue
August 30,2020

Disclaimer: The changes in the document may be accepted or rejected by the authors in their sole diseretion after our editing.However,sagesci is not responsible for revisions made to the document after our edit on **August30,2020**

sagesci Website:https://www.sagesci.cn/
2000+ native English editors:https://www.sagesci.cn/
